# Symptom-based Scoring for Acute Human Immunodeficiency Virus

**DOI:** 10.1093/cid/ciz059

**Published:** 2019-01-28

**Authors:** Eduard J Sanders, Alex Kigoro, Alexander Thiong’o, Eunice Nduati, Susan M Graham

**Affiliations:** 1 Kenya Medical Research Institute–Wellcome Trust Research Programme, Kilifi; 2 Nuffield Department of Medicine, University of Oxford, United Kingdom; 3 University of Washington, Seattle

To the Editor—We read with interest the report by Lin et al showing that a simple symptom score consisting of fever, myalgia, and weight loss accurately predicted acute human immunodeficiency virus (HIV) infection (AHI) [1]. We agree with the authors that symptom-based assessment is less prone to limitations inherent to risk-based scores, as “symptoms may be less subject to stigma, and therefore individuals may be more comfortable disclosing symptoms than sexual behaviors” [1]. Our concern is that the symptom score was developed in the United States, and therefore may not optimally identify AHI in resource-limited countries, as recommended by the authors.

We have previously developed a symptom-based score with data from at-risk and general populations from Kenya, Malawi, and South Africa [2]. This score assigns 1 point each for age 18–29 years or reported fever, fatigue, body pains, diarrhea, or sore throat, and 3 points for reported genital ulcer disease; individuals scoring ≥2 should be tested for AHI [2]. We are using this score to detect AHI with the Xpert HIV-1 Qual assay (Cepheid, Sunnyvale, California) among adults aged 18–39 years seeking urgent care in coastal Kenya (R01AI124968, ongoing). While HIV-1 RNA testing for AHI diagnosis is not supported by policy in sub-Saharan Africa, an exclusive focus on identifying chronic HIV in seropositive adults leads to missed opportunities [3]. This is especially important as preexposure prophylaxis (PrEP) is being scaled up in African settings.

The following case history from a voluntary testing and counseling center affiliated with our research clinic in coastal Kenya illustrates this: A 24-year-old heterosexual man tested negative on 2 HIV rapid antibody tests, whereas his female partner of 3 months tested antibody positive in the same session. He reported diarrhea and fatigue in the preceding 4 days, but no fever, weight loss, or myalgia. He was eligible for PrEP per Kenyan guidelines [4], as he was in a serodiscordant relationship. The patient met 3 of the criteria (young age, fatigue, and diarrhea) from our symptom-based score and was therefore tested with the Xpert HIV-1 Qual assay [2]. He tested positive, as confirmed by a viral load of 5500 copies/mL by Xpert HIV-1 Quant assay. He enrolled in an AHI cohort and started antiretroviral therapy shortly thereafter.

Per Kenyan guidelines, healthcare providers should assess for AHI symptoms prior to PrEP initiation when a recent high-risk exposure is reported [4]. While most front-line healthcare providers in sub-Saharan Africa received no specific training about AHI diagnosis [5, 6], PrEP guidelines offer a glimmer of hope that AHI symptoms will now be assessed in at-risk clients under evaluation for PrEP eligibility. We propose that this symptom screening should be done with our symptom-based score in African settings [2], as limiting AHI screening to those with fever, myalgia, and weight loss will lead to missed opportunities according to our data. As AHI testing should be targeted in resource-limited settings, we applaud the efforts of Lin and colleagues to promote the concept of targeted testing and encourage further research into this important area.

## Notes

***Financial support.*** This work was partially funded by the International AIDS Vaccine Initiative (IAVI) with the generous support of USAID and other donors; a full list of IAVI donors is available at www.iavi.org. The KEMRI–Wellcome Trust Research Programme is supported by core funding from the Wellcome Trust (grant number 203077/Z/16/Z). E. J. S. receives research funding from IAVI, the NIH (grant R01AI124968), and the Wellcome Trust. S. M. G. and E. J. S. were supported by the NIH (grant number R01AI124968). This work was also supported through the Sub-Saharan African Network for TB/HIV Research Excellence, a DELTAS Africa Initiative (grant number DEL-15-006).

***Potential conflicts of interest.*** All authors: No reported conflicts. All authors have submitted the ICMJE Form for Disclosure of Potential Conflicts of Interest. Conflicts that the editors consider relevant to the content of the manuscript have been disclosed.
